# Impact of Immunosuppression Type on 30-Day Postoperative Outcomes Following Anterior Cervical Discectomy and Fusion: A Retrospective National Cohort Study of 66,162 Patients

**DOI:** 10.1177/21925682261470356

**Published:** 2026-08-11

**Authors:** Rohan A. Phadke, Samer G. Salman, Connor Welsh, Nathan J. Lee

**Affiliations:** 1 School of Medicine, 3989 Baylor College of Medicine, Houston, TX, USA; 2 Midwest Orthopaedics at Rush, 12245 Rush University Medical Center, Chicago, IL, USA

**Keywords:** anterior cervical discectomy and fusion, immunosuppression, corticosteroids, biologic, 30-day outcomes, readmission

## Abstract

**Study Design:**

Retrospective Cohort Study.

**Objectives:**

Perioperative immunosuppression management in spine surgery lacks robust evidence. Most NSQIP-based ACDF models utilize a binary steroid variable, failing to distinguish corticosteroids from biologics or synthetic DMARDs. This study uses the granular NSQIP immunosuppression category and propensity score matching (PSM) to validate these risks.

**Methods:**

Primary ACDF patients were identified in ACS NSQIP (2015–2021; N=66,162). The primary exposure was preoperative corticosteroid use; a secondary analysis utilized the 2021 granular immunosuppression variable (n=9,761). The primary outcome was a 30-day composite adverse event (complication, readmission, reoperation, or mortality). Analysis included multivariable logistic regression and 1:3 PSM.

**Results:**

Preoperative corticosteroid exposure (3.8%) independently predicted the composite outcome (aOR 1.35, 95% CI 1.16–1.56), readmission (aOR 1.37), and medical complications (aOR 1.38; all p<0.001). Risk was driven by sepsis (aOR 2.00), UTI (aOR 1.67), and pneumonia (aOR 1.44), while wound complications and reoperation rates were not elevated. PSM confirmed these findings and revealed a mortality signal (aOR 2.10, p=0.03). Among 2021 patients, corticosteroids significantly increased risk (aOR 1.95; p=0.004), whereas biologic and synthetic DMARDs showed no significant elevation, though these subgroups were severely underpowered (<10%).

**Conclusions:**

Corticosteroid use is independently associated with increased 30-day infectious complications, readmission, and mortality following ACDF. Null findings for biologics and synthetic DMARDs reflect inadequate power rather than proven safety. Perioperative risk modification should prioritize minimizing corticosteroid burden over the cessation of biologic therapies.

## Introduction

Anterior cervical discectomy and fusion (ACDF), most commonly performed for cervical radiculopathy and myelopathy, is the most frequently performed cervical spine procedure in the United States.^1,2^ ACDF is generally considered safe and effective, with substantial symptom relief, low morbidity, and very low mortality.^3-5^ Despite these favorable outcomes, ACDF is increasingly performed in older and more medically complex patients, and postoperative complication rates have been reported as high as 16%.^4-9^ Given the high procedural volume of ACDF and its non-trivial postoperative complication burden, identifying modifiable or optimizable preoperative risk factors for complications, readmission, reoperation, mortality, non-home discharge, and prolonged length of stay remains clinically important.

Immunosuppression is an established perioperative risk factor in elective spine surgery, alongside patient factors such as age, diabetes, body mass index, and tobacco use.^10,11^ However, immunosuppression is a heterogeneous exposure. Patients may receive immunosuppressive therapy for autoimmune disease, inflammatory arthritis, malignancy, transplant history, or other systemic conditions, and treatment may include chronic corticosteroids, biologic disease-modifying antirheumatic drugs (DMARDs), conventional synthetic DMARDs, targeted synthetic agents such as Janus kinase inhibitors, or anti-rejection medications.^12-15^ These therapies differ substantially in mechanism of action, indication, reversibility, and perioperative management.^12-15^ Current perioperative guidelines increasingly reflect these distinctions, generally supporting continuation of conventional DMARDs, temporary withholding of biologic agents, and minimization of chronic corticosteroid exposure when clinically feasible.^16^ Therefore, grouping all immunosuppressive therapies may obscure clinically meaningful differences in perioperative risk.

The American College of Surgeons National Surgical Quality Improvement Program (ACS NSQIP) is a nationally validated, outcomes-based database commonly used for post-operative risk stratification.^17^ Several NSQIP-based ACDF risk models have evaluated predictors of adverse postoperative outcomes, but these studies have relied on the binary preoperative immunosuppression variable and have failed to distinguish corticosteroids from biologic or synthetic DMARDs.^18,19^ As a result, prior ACDF literature cannot determine whether the observed postoperative risk signal is driven primarily by corticosteroid exposure, other immunosuppressive medication classes, underlying disease severity, or the greater comorbidity burden of patients requiring these therapies. No published NSQIP ACDF study has used the granular immunosuppression category variable to evaluate medication-class-specific risk or applied propensity score matching to validate the steroid-associated risk signal.

The purpose of this study was to evaluate the association between preoperative corticosteroid use and 30-day postoperative outcomes following primary ACDF using a large national ACS NSQIP cohort from 2015 to 2021. We further sought to determine whether postoperative risk differed across immunosuppression categories, including corticosteroids, biologic DMARDs, synthetic DMARDs, combination or other immunosuppression, and anti-rejection or transplant-related therapy. We hypothesized that preoperative corticosteroid use would be independently associated with higher odds of 30-day adverse outcomes after ACDF. By separating corticosteroid exposure from other immunosuppressive medication classes and validating the steroid-associated risk signal using multivariable adjustment and propensity score matching, this study aimed to clarify whether perioperative risk modification after ACDF should focus primarily on corticosteroid burden rather than generalized immunosuppression alone.

## Methods

### Data Source and Study Population

We conducted a retrospective cohort study using the American College of Surgeons National Surgical Quality Improvement Program (ACS NSQIP) Participant Use Files from 2015 to 2021. Patients undergoing ACDF were identified using Current Procedural Terminology (CPT) codes 22551 or 22554, with or without the multilevel add-on code 22552. Patients were excluded if they were younger than 18 years, designated as American Society of Anesthesiologists (ASA) physical status class V, flagged as emergency cases, or had concurrent posterior cervical fusion. Prior to analysis, duplicate records arising from overlapping Participant Use File release years were identified by the unique case identifier and removed, retaining one record per surgical encounter.

### Exposure Variables

The primary exposure was preoperative corticosteroid use, recorded as a binary variable in the NSQIP dataset. A secondary analysis used the immunosuppression category variable, available in NSQIP from 2021 onward, which classifies patients into six mutually exclusive groups: no immunosuppression, corticosteroids only, synthetic disease-modifying antirheumatic drugs (DMARDs) only, biologic DMARDs only, combination or other regimens, and anti-rejection or transplant medications. This secondary cohort comprised 9,761 patients.

### Outcome Measures

The primary outcome was a composite 30-day adverse event, defined as any postoperative complication, unplanned readmission, unplanned reoperation, or 30-day mortality. Secondary outcomes included individual complication subtypes (systemic sepsis, urinary tract infection [UTI], pneumonia, surgical site infection [SSI], thromboembolic events, and cardiac or neurologic complications), non-home discharge, and total hospital length of stay.

### Statistical Analysis

Baseline characteristics were compared between groups using independent-samples t-tests for continuous variables and chi-square tests for categorical variables. Primary multivariable analysis used logistic regression adjusted for 19 covariates: age, sex, body mass index (BMI), ASA classification, functional dependency, procedure level, diabetes, tobacco use, hypertension, chronic obstructive pulmonary disease (COPD), congestive heart failure (CHF), dialysis dependence, disseminated malignancy, significant weight loss, bleeding disorder, preoperative albumin below 3.5 g/dL, operative time, the 5-item Modified Frailty Index (mFI-5), and operative year.

Propensity score matching (PSM) served as the primary sensitivity analysis. A propensity score for corticosteroid exposure was estimated from the same covariates using logistic regression. Patients were matched 1:3 (steroid to control) by nearest-neighbor matching with a caliper of 0.2 standard deviations of the logit-transformed propensity score. Balance was assessed using standardized mean differences (SMDs), with values below 0.10 indicating adequate balance. Length of stay was analyzed using negative binomial regression and reported as an incidence rate ratio (IRR). Subgroup analyses used Wald tests for product interaction terms in the full multivariable model. Post-hoc power calculations for sparse immunosuppression category subgroups used a two-proportions z-test. All analyses were conducted in Python using the statsmodels and scipy libraries.

## Results

### Study Cohort and Baseline Characteristics

Of 66,162 patients undergoing ACDF between 2015 and 2021, 2,499 (3.8%) had documented preoperative corticosteroid use. Baseline characteristics differed substantially between groups (Table 1). Steroid-exposed patients were more often female (57.9% vs 49.4%), older (mean age 58.9 vs 55.7 years), and carried a significantly higher comorbidity burden, including higher rates of COPD (9.1% vs 4.5%), hypertension (56.4% vs 47.6%), CHF (1.4% vs 0.5%), and functional dependency (3.2% vs 1.8%). Operative time and procedure level were similar between the two groups.

**Table 1.** Baseline Characteristics by Preoperative Corticosteroid Use, ACS NSQIP 2015-2021 (N=66,162)

Variable	Corticosteroid use (n=2,499)	No corticosteroid (n=82,566)	p-value
**Demographics**			
Age, mean (SD), years	58.9 (11.4)	55.7 (11.7)	<0.001
Female sex	1,446 (57.9%)	31,448 (49.4%)	<0.001
BMI, mean (SD), kg/m2	30.7 (7.1)	30.7 (6.6)	0.93
**Comorbidities**			
Diabetes mellitus	503 (20.1%)	11,326 (17.8%)	0.003
Hypertension requiring medication	1,409 (56.4%)	30,279 (47.6%)	<0.001
Current smoker	486 (19.4%)	15,856 (24.9%)	<0.001
COPD	228 (9.1%)	2,850 (4.5%)	<0.001
Congestive heart failure	34 (1.4%)	315 (0.5%)	<0.001
Dialysis-dependent renal failure	17 (0.7%)	184 (0.3%)	<0.001
Disseminated cancer	24 (1.0%)	105 (0.2%)	<0.001
Bleeding disorder	59 (2.4%)	752 (1.2%)	<0.001
Weight loss >10% in 6 months	8 (0.3%)	111 (0.2%)	0.15
**Functional and Nutritional Status**			
Functionally dependent (partial or total)	79 (3.2%)	1,088 (1.7%)	<0.001
Albumin <3.5 g/dL	137 (5.5%)	1,777 (2.8%)	<0.001
mFI-5, mean (SD)	0.9 (0.8)	0.7 (0.8)	<0.001
ASA class III-IV	1,625 (65.0%)	30,481 (47.9%)	<0.001
**Operative Characteristics**			
Multilevel procedure	1,246 (49.9%)	31,154 (48.9%)	0.38
Operative time, mean (SD), min	128.9 (63.4)	128.1 (65.9)	0.50
Transfusion (intraoperative)	6 (0.24%)	0 (0.06%)	0.004
Wound class I (clean)	99.3%	99.4%	NS
**30-Day Outcomes**			
Composite adverse outcome	225 (9.0%)	3,424 (5.4%)	<0.001
Any medical complication	113 (4.5%)	1,590 (2.5%)	<0.001
Unplanned readmission	134 (5.4%)	2,030 (3.2%)	<0.001
Reoperation	50 (2.0%)	1,012 (1.6%)	0.13
30-day mortality	16 (0.6%)	142 (0.2%)	<0.001
Wound/SSI composite	20 (0.8%)	380 (0.6%)	0.25
Non-home discharge	221 (8.8%)	3,565 (5.6%)	<0.001
Median length of stay, days (IQR)	1 (1-2)	1 (1-2)	0.002*

BMI=body mass index; COPD=chronic obstructive pulmonary disease; mFI-5=5-item modified frailty index; ASA=American Society of Anesthesiologists; SSI=surgical site infection; IQR=interquartile range.

^*^LOS p-value from negative binomial regression (IRR 1.06, 95% CI 1.02-1.10). Continuous variables by t-test; categorical by chi-square.

### Primary Outcomes: Corticosteroid Use and 30-Day Complications

**Figure 1.**
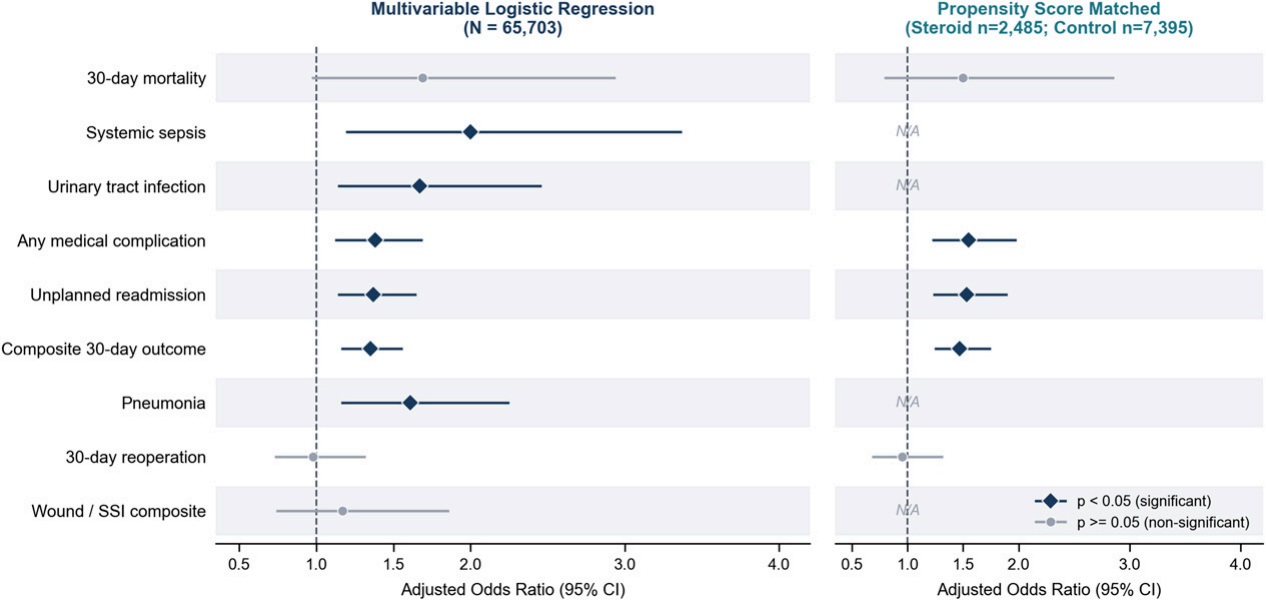
Adjusted odds ratios for steroid use: Multivariable and propensity score matched analyses. Dual-panel forest plot. Left panel: adjusted odds ratios (aOR) from multivariable logistic regression (N=65,703). Right panel: PSM-matched analysis (steroid n=2,485, controls n=7,395; 1:3 nearest-neighbor matching; all covariate SMD <0.10 post-matching). Diamond markers = p<0.05; circular markers = non-significant. Steroid use significantly predicted medical complications (sepsis aOR 2.00, UTI aOR 1.67, pneumonia aOR 1.61, any complication aOR 1.38) and readmission (aOR 1.37), but not wound/SSI or reoperation. PSM confirmed composite findings and revealed a significant mortality signal (aOR 2.10, p=0.03) not evident in the primary model. N/A = subtype-specific PSM not performed. aOR=adjusted odds ratio; PSM=propensity score matched; SMD=standardized mean difference; SSI=surgical site infection; UTI=urinary tract infection. Reference = no properative corticosteroid use. Adjusted for age, sex, BMI, ASA class, functional dependency, procedure level, diabetes, smoking, hypertension, COPD, CHF, dialysis, malignancy, weight loss, bleeding disorder, albumin <3.5 g/dL, operative time, mFI-5, and operative year. PSM: 1:3 nearest-neighbor matching; all covariates balanced (SMD<0.10). N/A = PSM analysis not run for individual complication subtypes

Unadjusted composite 30-day adverse event rates were significantly higher in steroid-exposed patients (9.0% vs 5.4%; p<0.001). After full covariate adjustment, preoperative corticosteroid use remained an independent predictor of the composite outcome (adjusted odds ratio [aOR] 1.35, 95% confidence interval [CI] 1.16-1.56; p<0.001), unplanned readmission (aOR 1.37, 95% CI 1.14-1.65), and any medical complication (aOR 1.38, 95% CI 1.12-1.65; all p<0.001). Full outcome results are presented in Table 2 and Figure 1.

**Table 2.** Multivariable Logistic Regression and Propensity Score Matched Analysis: Effect of Corticosteroid Use on 30-Day Outcomes After ACDF (N=65,703)

Outcome	Steroid (n=2,499) n (%)	No Steroid (n=63,663) n (%)	Multivariable aOR (95% CI)	p	PSM-Matched aOR (95% CI)	p
**Individual Outcomes**						
30-day mortality	16 (0.6%)	142 (0.2%)	1.69 (0.97-2.94)	0.062	1.50 (0.79-2.86)	0.22
Systemic sepsis	17 (0.7%)	148 (0.2%)	**2.00 (1.19-3.37)**	**0.009**	**--**	--
Urinary tract infection	30 (1.2%)	330 (0.5%)	**1.67 (1.14-2.46)**	**0.009**	**--**	--
Pneumonia	42 (1.7%)	446 (0.7%)	**1.61 (1.16-2.25)**	**0.005**	**--**	--
Any medical complication	113 (4.5%)	1,590 (2.5%)	**1.38 (1.12-1.69)**	**0.002**	**1.55 (1.22-1.98)**	**<0.001**
Unplanned readmission	134 (5.4%)	2,030 (3.2%)	**1.37 (1.14-1.65)**	**<0.001**	**1.53 (1.23-1.90)**	**<0.001**
30-day reoperation	50 (2.0%)	1,012 (1.6%)	0.98 (0.73-1.32)	0.0	0.95 (0.68-1.32)	0.74
Wound/SSI composite	20 (0.8%)	380 (0.6%)	1.17 (0.74-1.86)	0.49	--	--
**Secondary Outcomes**						
Non-home discharge	221 (8.8%)	3,565 (5.6%)	1.14 (0.97-1.34)	0.10	--	--
Length of stay (IRR)	--	--	**1.06 (1.02-1.10)**	**0.002**	--	--
**Summary**						
Composite 30-day outcome	225 (9.0%)	3,424 (5.4%)	**1.35 (1.16-1.56)**	**<0.001**	**1.47 (1.24-1.75)**	**<0.001**

aOR=adjusted odds ratio; IRR=incidence rate ratio; PSM=propensity score matched; SSI=surgical site infection; --=not applicable or not run for individual subtypes. Reference: no preoperative corticosteroid use. Multivariable model adjusted for age, sex, BMI, ASA class, functional dependency, procedure level, diabetes, smoking, hypertension, COPD, CHF, dialysis, malignancy, weight loss, bleeding disorder, albumin <3.5 g/dL, operative time, mFI-5, and operative year. PSM: 1:3 nearest-neighbor; all SMD <0.10 post-matching. Bold values indicate statistical significance (p<0.05). IRR from negative binomial regression for length of stay.

### Individual Complication Subtypes

Stratification by complication type demonstrated that the excess risk was driven by infectious and medical complications rather than surgical ones (Table 3, Figure 1). Systemic sepsis was approximately twice as likely in steroid-exposed patients (aOR 2.00, 95% CI 1.19-3.37; p=0.009), as were urinary tract infections (aOR 1.67, 95% CI 1.14-2.46; p=0.001) and pneumonia (aOR 1.61, 95% CI 1.16-2.25; p=0.020). In contrast, wound complications and SSIs were not significantly elevated (aOR 1.17, 95% CI 0.74-1.86; p=0.49), nor was the 30-day reoperation rate (aOR 0.98, 95% CI 0.73-1.32; p=0.90).

**Table 3.** Individual 30-Day Complication Subtypes by Preoperative Corticosteroid Use (N=65,703)

Complication	Steroid (%)	No Steroid (%)	Adjusted OR (95% CI)	p-value
**Infectious complications**				
Systemic sepsis	0.68	0.23	**2.00 (1.19-3.37)**	**0.009**
Urinary tract infection	1.20	0.52	**1.67 (1.14-2.46)**	**0.009**
Pneumonia	1.68	0.70	**1.61 (1.16-2.25)**	**0.005**
**Wound/surgical site**				
Superficial SSI	0.28	0.30	<10 events	NS
Deep incisional SSI	0.20	0.11	1.55 (0.61-3.93)	0.35
Organ space SSI	0.24	0.15	1.12 (0.47-2.64)	0.80
Wound dehiscence	0.08	0.05	<10 events	NS
**Thromboembolic**				
Deep vein thrombosis	0.28	0.28	0.75 (0.35-1.64)	0.47
Pulmonary embolism	0.28	0.23	0.92 (0.42-1.99)	0.83
**Cardiac/neurologic**				
Cardiac arrest	0.16	0.14	0.80 (0.29-2.22)	0.67
Stroke/CVA	0.08	0.08	<10 events	NS
Reintubation	0.72	0.49	0.96 (0.58-1.59)	0.88
**Mortality**				
30-day mortality	0.64	0.22	1.69 (0.97-2.94)	0.062

SSI=surgical site infection; CVA=cerebrovascular accident. Reference: no preoperative corticosteroid use. Adjusted for age, sex, BMI, ASA class, functional dependency, procedure level, diabetes, smoking, hypertension, COPD, CHF, dialysis, malignancy, weight loss, bleeding disorder, albumin <3.5 g/dL, operative time, mFI-5, and operative year. <10 events: model not fit due to insufficient events. Bold values indicate statistical significance (p<0.05). Note: 30-day mortality reached significance on PSM analysis (aOR 2.10, 95% CI 1.09-4.03; p=0.03).

### Propensity Score Matched Analysis

Propensity score matching yielded 2,485 steroid-exposed patients matched to 7,395 controls, with all covariate SMDs falling below 0.10 after matching. The matched analysis confirmed the primary multivariable findings, with a composite adverse event aOR of 1.47 (95% CI 1.24-1.75; p<0.001). 30-day mortality did not reach statistical significance in either the primary logistic model (aOR 1.69, 95%CI 0.97-2.94; p=0.062) or the propensity-matched analysis (aOR 1.50, 95% CI 0.79-2.86; p=0.22), as shown in Table 2 and Figure 1. The absolute rate difference was small (0.6% vs 0.2%; 16 events in the steroid group), and given the modest event count this finding should be interpreted with caution.

### Subgroup Analyses and Effect Consistency

**Figure 2.**
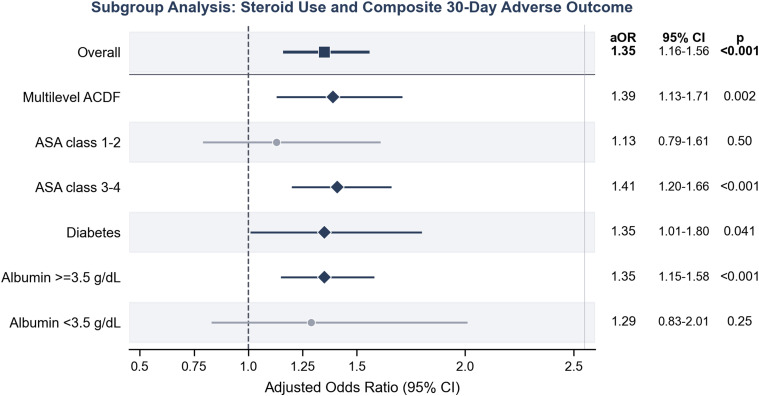
Subgroup analysis: Consistency of steroid risk across clinical strata. Forest plot of adjusted odds ratios for 30-day composite adverse outcome in steroid-exposed vs non-exposed patients across pre-specified subgroups. The overall estimate (aOR 1.35) is shown as a square marker; subgroups as diamonds (significant) or circles (non-significant). p-interaction values are from Wald tests of steroid x subgroup interaction terms in the full multivariable model; all were non-significant (p>0.05), indicating consistent effect size across procedure level, ASA class, diabetes status, and albumin level. The albumin <3.5 g/dL subgroup was underpowered (n=170 steroid-exposed). Single-level-only and no-diabetes subgroups not shown due to model convergence issues (stratifying variable collinear). aOR=adjusted odds ratio; ASA=American Society of Anesthesiologists. All subgroup models adjusted for age, sex, BMI, functional dependency, comorbidities, operative time, mFI-5, and operative year (stratifying variable excluded). P-int: Wald test p-value for steroid x subgroup interaction term in the combined multivariable model

The steroid effect on the composite outcome was consistent across all pre-specified subgroups, including procedure level (single vs multilevel), ASA class (I-II vs III-IV), presence of diabetes, and preoperative albumin level. No significant interaction was identified for any subgroup variable (all p-interaction >0.05), supporting a homogeneous treatment effect across clinically distinct patient populations (Figure 2).

### Secondary Outcomes

Steroid-exposed patients had a modest but statistically longer hospital stay compared to non-exposed patients (IRR 1.06, 95% CI 1.02-1.10; p=0.002; Table 2); however, median length of stay was identical at 1 day (IQR 1-2) in both groups, and the small adjusted difference is unlikely to be clinically meaningful. Non-home discharge rates did not differ significantly after adjustment (aOR 1.14, 95% CI 0.97-1.34; p=0.10).

### Immunosuppression Category Analysis

**Figure 3.**
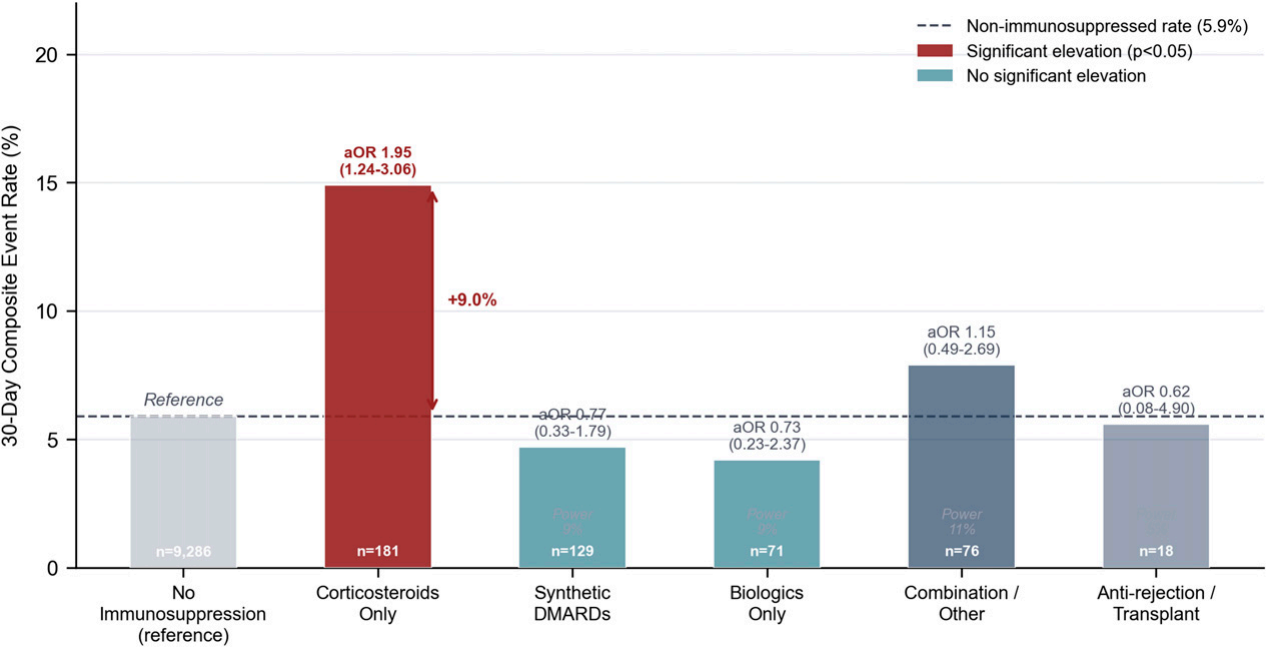
30-day composite event rates by immunosuppression category (2021 NSQIP Cohort). 2021 NSQIP cohort only (immunosuppression category variable). Composite 30-day = any complication, unplanned readmission, reoperation, or mortality. Adjusted ORs from multivariable logistic regression (age, sex, BMI, ASA class, procedure level, diabetes, smoking, hypertension, operative time, mFI-5). Observed post-hox power shown for underpowered null-finding groups. Required n=1,451/group to detect OR=1.5. Crude 30-day composite adverse event rates stratified by immunosuppression category (2021 ACS NSQIP; N=9,761). Dashed reference line = non-immunosuppressed rate (5.9%). Adjusted ORs are annotated above each bar. Corticosteroids-only patients (n=181) had a significantly elevated rate (14.9%; aOR 1.95, p=0.004). Biologic DMARD (n=71, aOR 0.73) and synthetic DMARD (n=129, aOR 0.77) groups showed no significant elevation; however, observed power was under 10% for both groups. Required n=1,451/group to detect OR 1.5. DMARD=disease-modifying antirheumatic drug; aOR=adjusted odds ratio

Among the 9,761 patients in the 2021 cohort with immunosuppression category data, corticosteroids-only patients (n=181) had a crude composite event rate of 14.9%, nearly three times the reference rate of 5.9% among non-immunosuppressed patients (Figure 3). After adjustment, this group retained a significantly elevated risk (aOR 1.95, 95% CI 1.24-3.06; p=0.004; Table 4). Biologic DMARD patients (n=71) and synthetic DMARD patients (n=129) showed no statistically significant elevation in risk (aOR 0.73 and 0.77, respectively). Post-hoc power calculations demonstrated that these subgroups had fewer than 10% observed power to detect an odds ratio of 1.5, and a minimum of 1,451 patients per group would be required to achieve 80% power at that effect size.

**Table 4.** 30-Day Outcomes by Immunosuppression Category: 2021 NSQIP Cohort (N=9,761)

Category	n	Composite 30-day (%)	SSI/Wound (%)	Readmit (%)	Reop (%)	Adjusted OR (95% CI)	p	Observed power
No immunosuppression (ref)	9,286	5.9	0.7	3.5	1.7	Reference	​	N/A
Corticosteroids only	181	14.9	1.1	8.3	4.4	**1.95 (1.24-3.06)**	**0.004**	N/A
Synthetic DMARDs only	129	4.7	0.8	2.3	1.6	0.77 (0.33-1.79)	0.55	8.9%
Biologic DMARDs only	71	4.2	0.0	4.2	0.0	0.73 (0.23-2.37)	0.60	9.3%
Combination/other	76	7.9	1.3	5.3	0.0	1.15 (0.49-2.69)	0.75	11.4%
Anti-rejection/transplant	18	5.6	0.0	0.0	5.6	0.62 (0.08-4.90)	0.65	5.0%

DMARD=disease-modifying antirheumatic drug; Immunosuppression category data available in NSQIP from 2021 onward only. Adjusted ORs from multivariable logistic regression (age, sex, BMI, ASA class, procedure level, diabetes, smoking, hypertension, operative time, mFI-5). Reference=no immunosuppression. Observed power: post-hoc two-proportions z-test for detecting the observed rate vs reference, n per group. Required n=1,451 per group to detect OR 1.5 (alpha=0.05, power=80%) and n=445 to detect OR 2.0. Null findings for biologic and synthetic DMARD groups must be interpreted as underpowered, not as evidence of safety. Bold red values = statistically significant (p<0.05).

## Discussion

This study demonstrates that preoperative corticosteroid use is independently associated with increased 30-day medical complications and unplanned readmission following primary ACDF in a large national cohort of 66,162 patients. Importantly, the excess risk was driven by infectious and systemic complications, sepsis, urinary tract infection, and pneumonia, rather than surgical site infections or reoperation. These findings were robust across multivariable logistic regression and propensity score–matched analyses, and the steroid-associated risk was consistent across all pre-specified subgroups, including procedure level, ASA class, diabetes status, and preoperative albumin level. Notably, the steroid-associated risk did not differ significantly by procedure level (no significant steroid-by-level interaction, p=0.93), indicating that the signal is not confined to the older, multilevel population in whom corticosteroid use most often reflects chronic immunosuppression rather than a brief acute course.

The infectious rather than surgical nature of the complication signal is consistent with the known immunologic effects of chronic corticosteroid exposure, which broadly suppresses both innate and adaptive immunity, impairing neutrophil function, T-cell–mediated responses, and mucosal barrier integrity. This pattern mirrors findings in posterior lumbar fusion, where Kebaish et al demonstrated that long-term corticosteroid use was independently associated with increased infection, readmission, and reoperation after propensity matching in over 140,000 NSQIP patients.^20^ Similarly, Singla et al reported that chronic oral steroid use was associated with a 1.74-fold increased risk of surgical site infection and a 2.06-fold increased risk of 1-year mortality following lumbar fusion in Medicare beneficiaries.^21^ The present study extends these findings to the ACDF population and further clarifies that the risk signal is predominantly medical rather than wound-related.

The finding that most readmissions after ACDF are driven by nonsurgical complications is well established. Samuel et al reported that 53.8% of 30-day readmissions after ACDF were for nonsurgical site–related reasons, with pneumonia and cardiovascular events among the most common causes.^22^ Zaki et al similarly identified systemic infection and sepsis as the leading cause of readmission after ACDF.^23^ The present study adds to this literature by identifying chronic corticosteroid exposure as a modifiable contributor to these medical readmissions, suggesting that perioperative risk stratification and targeted infection prevention strategies may be warranted in this population. The development and implementation of robust predictive models, could facilitate this risk stratification by identifying patients who require the most intensive preoperative optimization.^24,25^ These efforts align with the broader goals of quality improvement and value-based care in spine surgery. ^26^

A key contribution of this study is the use of the NSQIP immunosuppression category variable, available from 2021, to distinguish corticosteroid exposure from biologic and synthetic DMARD use. The corticosteroids-only subgroup demonstrated a nearly twofold increase in composite adverse events (aOR 1.95), whereas biologic and synthetic DMARD groups showed no statistically significant elevation. However, these null findings must be interpreted with extreme caution: post-hoc power calculations demonstrated fewer than 10% observed power to detect an odds ratio of 1.5, and a minimum of 1,451 patients per group would be required to achieve 80% power at that effect size. These results, therefore, reflect an inadequate sample size rather than a confirmed absence of risk. This distinction is clinically important, as premature reassurance regarding DMARD safety could influence perioperative decision-making.

These findings have implications for perioperative immunosuppression management. The 2022 ACR/AAHKS guideline conditionally recommends continuing conventional synthetic DMARDs through surgery and withholding biologic DMARDs for one dosing interval before elective total joint arthroplasty.^14^ Glucocorticoids have consistently been identified as the strongest pharmacologic risk factor for postoperative infection across surgical specialties, and current guidelines emphasize minimizing corticosteroid burden when clinically feasible. The present data support this approach in the ACDF population: perioperative risk modification should focus on corticosteroid exposure rather than reflexive cessation of biologic therapy, which may precipitate disease flares without clear perioperative benefit. A systematic review by van Duren et al found that continuing biologic DMARDs perioperatively was associated with a significantly lower risk of disease flares without a significant increase in surgical site infections or wound complications, further supporting a targeted rather than blanket approach to immunosuppression management.^27^ Although the synthetic and biologic DMARD subgroups showed no increased infectious risk, these estimates were severely underpowered (<10% power) and cannot, in isolation, justify modifying perioperative DMARD-withholding practices; adequately powered prospective study is required before national recommendations are revised.

### Limitations

Several limitations should be acknowledged. The NSQIP corticosteroid variable is binary and does not capture dose, duration, or indication, precluding distinction between chronic high-dose immunosuppression and brief perioperative dexamethasone. Confounding by indication remains the most important residual concern, as patients requiring long-term corticosteroids likely have more severe underlying systemic disease than is captured by available covariates. The NSQIP dataset lacks a dedicated rheumatologic disease comorbidity variable, and the immunosuppression category variable is available only from 2021, substantially limiting the temporal scope and statistical power of subgroup analyses. Finally, NSQIP captures outcomes through 30 days, which may underestimate longer-term infectious or wound complications relevant to immunosuppressed patients. The binary corticosteroid variable also cannot distinguish the brief acute-course exposure more common in younger single-level patients from the chronic immunosuppression more typical of older multilevel disease, populations that likely differ in baseline risk.

### Future Directions

Several avenues for future investigation emerge from this work. First, prospective studies or analyses using datasets that capture corticosteroid dose, duration, and indication are needed to establish whether a dose-response relationship exists and to identify a clinically meaningful steroid burden threshold above which perioperative risk is significantly elevated. Second, as the NSQIP immunosuppression category variable accumulates additional years of data, adequately powered analyses of biologic and synthetic DMARD subgroups will become feasible, enabling definitive assessment of medication-class-specific risk after ACDF. Third, the development of spine-specific perioperative immunosuppression guidelines is warranted; current recommendations are extrapolated primarily from the arthroplasty literature, and the distinct surgical environment, fusion biology, and complication profile of cervical spine surgery may necessitate tailored protocols. Fourth, future studies should evaluate whether targeted perioperative interventions, such as preoperative steroid tapering, enhanced infection surveillance, or antimicrobial prophylaxis optimization, can mitigate the excess infectious risk identified in this cohort. Exploring minimally invasive alternatives may further mitigate risk in these vulnerable populations.^28^ Finally, linkage of NSQIP data with disease-specific registries or claims databases would allow adjustment for underlying rheumatologic or autoimmune disease severity, addressing the residual confounding by indication that limits all current administrative database analyses.

## Conclusion

Preoperative corticosteroid use is independently associated with higher 30-day medical complications, unplanned complications and unplanned readmission following primary ACDF. The excess risk is infectious rather than surgical in nature, driven by sepsis, urinary tract infection, and pneumonia. Biologic and synthetic DMARD null findings are severely underpowered and should not be interpreted as evidence of safety. These results suggest that perioperative risk modification in ACDF patients on immunosuppressive therapy should focus on corticosteroid burden rather than blanket cessation of biologic or synthetic DMARD therapy. Adequately powered, prospective studies with granular medication data are needed to refine medication-class-specific risk estimates and inform the development of spine-specific perioperative immunosuppression guidelines.

## Data Availability

NSQIP ACS participant use file data is privately available to participating hospitals; however, data used may be made available to the journal and editors upon reasonable request.*
